# Dissertatio Medica Inauguralis *De Dysenteria Regionum Calidarum*

**Published:** 1818-03

**Authors:** 


*Dissertatio Medica Inauguralis* DE DYSENTERIA REGIONUM CALIDARUM. Auctore ARCHIBALDO ROBERTSON, Societ: Reg: Med: Edin: Sodal: et in Classe Regiâ Chirurg:—*pp.* 48. *Edinburgi.* 1817.

IN the different universities of Europe which confer medical degrees—other than honorary,—it is well known that one of the qualifications required in the candidate for the honours of the Doctorate, is the composition, or at least presentation, of an Essay or Thesis on some medical subject, or on some subject connected, more or less remotely, with the collateral branches of science and phylosophy. Essential, however, as the Essay itself is to the acquisition of the honour, it is generally believed that its *character* is seldom or ever taken as an item in the calculations which determine the fitness or unfitness of the candidate for the honours to which he aspires. This fitness or unfitness is ascertained by other trials, (which are, it must be allowed, sufficiently strict and severe at the British universities); and, provided these have been found satisfactory, it is well understood that the qualities of the Thesis have little influence either in obstructing the ascent or smoothing the way, to the wished for dignity. A little vain triumph or disgrace may indeed give a transient shock to the nerves of the nascent physician on the final day of his academic labours—while receiving the public praise or censure of his essay from the medical faculty;—yet if the composition be of the statute length,—be tolerably free from grammatical errors—and contain no doctrine very particularly absurd,—it is generally permitted to be subjected "eruditorum examini"—even although it should chance to be framed of a language which Celsus, or even Paracelsus, might find no slight difficulty in perusing, and contain doctrines which Cullen or Darwin would find no easy matter to understand,—and exhibit a species of reasoning which Bacon or Stewart would scarcely recognize as appertaining to their favourite science. The consequence of this, as might naturally be expected from the careless indolence of human nature, is, that the majority of these initiatory essays are, in truth, very little creditable either to their authors, or to the medical school whence they originate. To this, however, it must be allowed, there are many exceptions. Fortunately for the general character of these productions, there are principles in human nature which operate independently of statute, and lead minds that are truly noble, to act well and worthily, when bound by no ties either of law or fashion. Ambition — the desire of being distinguished—the wish to gain the good opinion of friends or even strangers—or perhaps the innate buoyancy of talent —leads many candidates to leave the beaten track. From minds of this cast have sprung Dissertations equally honourable to themselves and to their school,—worthy to be placed among those productions which adorn the shelves of the most intelligent physicians of the present day, and capable of rendering their authors known to posterity. Of this class we could now enumerate not a few which have issued from that school with which we are best acquainted (Edinburgh) within the last forty years, many of which are, indeed, well known to the public in the new and enlarged form, which the notice they attracted in their original dress induced their authors subsequently to give them. Of these *good Theses,* which we recollect to have consulted in former years in the university library, the greater number belonged to one or other of the following classes: 1st. Some Original Physiological Speculations; 2d. Some Experimental Enquiry; 3d. Good Epitome of the actual State of our Knowledge of some Disease, or other scientific subject; and, lastly, The Detail of some Subject of Practice from the Author's own Experience.

This last species of excellence is, for very obvious reasons, the rarest, and is only to be looked for in the compositions of those medical men, who, after being established in practice, have once more returned to school. Of this class of graduates the majority, we believe, has always been furnished by the medical departments of the Army and Navy; and we have been informed that (as might be expected) the supply from these sources has been much augmented since the return of peace. This, indeed, appears very evidently to be the case from the list of graduations at Edinburgh, given in our number for September; as our readers will have perceived that, of the ninety two candidates that assumed the doctorial bonnet in the last season, more than one fourth of the number were officers in the public service. This is certainly highly creditable to these gentlemen, and augurs most favourably of the general character of progressive improvement by which the medical department of our fleets and armies are so eminently distinguished. And it is no doubt to this circumstance that we are to attribute a novelty which has struck us, for several years past, in the annual lists of Edinburgh graduations; we allude to the unusual prevalence of subjects of a practical nature in the titles of the inaugural dissertations.

Of the general character or particular value of these dissertations it is out of our power to give any account, as, for very obvious reasons, it can rarely be our lot to meet with them. We have however lately been favoured by a friend with a few of these academic exercises,—with the general character of which, as well as with the general arcana of the doctorate, we may perhaps at some future period favour our readers. In the mean time we propose laying before them a detailed analysis of one of the most valuable of those which have issued from the Edinburgh university press this year, viz. Dr. Robertson's, the title of which is prefixed to this article.

This little Essay is indeed extremely creditable to the author, and in point of elegance of composition and classical purity of style, needs not shrink from a comparison with our best modern medical writers of latin. Of the justness of this opinion our readers will be able to judge from the extracts we shall give from the essay itself. With regard to the pathological and practical doctrines contained in it, we may observe that they are, upon the whole, in themselves, so sensible and ingenious, and so consonant with those maintained by ourselves in different parts of these our labours, as to run little risk of our reprehension: they are, moreover, presented to us in so graceful a form and with so much ingenuous modesty, that we should be more than even *critically* severe, did we visit with our most unpitying perspicacity this fair — but certainly not faultless production.

After a short but well written Introduction, from which we learn that our author's experience of the disease of which he treats was principally obtained, during his service as surgeon of a frigate, in the unfortunate expedition to New Orleans in 1815,—the history of tropical dysentery in its more ordinary form is clearly and concisely detailed. The Doctor remarks that he never saw scybala in this disease; he also states the accompanying fever to have been occasionally of the remittent type. In the following pages (6—7) he describes the disease in its more violent form, and with the peculiarities which it assumed in the Mississippi. We shall give the author's own words.

"Primò aeger languet, et appetitum cibi simul cum animi hilaritate penitùs amittit. Male se habet, sed quod sibi dolet dicere nescit. Nonnunquam de dolore obtuso regionem hepatis infestante, digitis prementibus aucto, et eructatione paulispèr levato, conqueritur; sed dolor iste vix est ullus, aut saltèm opinione multò minor, si quis illum aestimare licuerit ex hepatis conditione post mortem repertâ. Indicii hujusce praesentia characterem morbi verum denotat; sed in exemplis plurimis omninò abest; hinc obscuritas major. Re verâ indagator etiam solertissimus nullum adversæ valetudinis signum praeter linguam albidam et pulsum justo frequentiorem (circiter nonaginta sex), detegere possit.

"Res ità per quatuor, sex, vel octo dies se habere solent, et medicus, cui deest regionum calidarum experientia, morbum levem, periculi expertem et sanatu facilem, fortasse praedicabit—sed perperàm; nam functiones magis magisque a statu sano quotidie abeunt; Pyrexia latens, insidiosa, gradibus vix percipiendis infelicem terit, et demùm Dysenteriae indicia vera, nempè tenesmus et dejectiones penè innumerae, violentiâ maximâ erumpunt. Dehinc omnia in pejus ruunt; febris ardet, adest delirium, sudor quoque oleosus manat, ventriculum vomitus vexat, anxietas febrilis sitisque urgent, et lingua strato obducitur flavo versus finem morbi in crustam nigram immobilem desituro. Copia magna materiae viridis biliosae et sanguineae ex alvo profluit, et medico tandèm persuadet morbidum hepatis atque bilis statum ferè semper alvi flûxus irritamentum esse. Hìc tormina leviora vel penè nulla sunt, sed interdùm tenesmus infestissimus diù trahens, ex ano portionem intestini recti, more visu horribili, detrudit. Deincèps instant subsultus tendinum, sudores frigidi, et singultus, et nisi priùs aeger plurimùm sublevetur quàm hi emicaverint, de eo paucis horis actum erit."

The following were the morbid appearances observed by Dr. Robertson in these cases:— Inflammation of the liver invariably;—this viscus almost always greatly enlarged, generally containing in its substance one or more abscesses of a watery pus, which, in three or four instances amounted to thirty ounces! Nothing remarkable in the gall bladder, but the internal coats of the alimentary canal exhibited, in every case, ulcerations of a greater or less extent,—particularly the colon and rectum. In chronic cases the liver, if it did not always contain pus, was always greatly enlarged and indurated. The spleen and pancreas were sometimes, though rarely, similarly affected; so also were the mesenteric glands.

Of the next section, which is entitled "De Pathologia et Ætiologia," and which contains a good deal of novelty and ingenious pathological reasoning, we shall lay the subject before our readers in our own words;—apologising to the author for the injury which cannot fail being done to his Essay by our hasty and extemporaneous translation. "That faculty of the living animal body by which it is enabled to preserve a nearly equable degree of temperature amid all the variations of the external air, is known to every one: the manner however in which this faculty operates in the production of the event is very imperfectly known; and it will perhaps be wise for us still to consider this as one of Nature's inscrutable secrets, until we shall have become better acquainted with the nature of the vital principle,—with the operation of which the phænomenon in question seems so intimately connected.—In northern climates, the medium temperature of which may be placed at about the 50th degree of Fahrenheit, it is obvious, that in order to preserve the established temperature of the human body, a portion of free caloric must be continually evolved, amounting to no less than 47° of the same scale. Without this steady and certain production of heat, no extraneous means, to which recourse might be had, could preserve the solids and fluids in a condition fit to discharge the various functions necessary to life. In the event of change of climate, it is very probable, that the vital principle, for some time, continues to produce the same quantity of caloric as before;—that, for example, the body after being transported into a tropical climate, where the mean atmospheric temp. is about 80°, continues for a certain period at least, to generate as much caloric as it was accustomed to do in the mean temp. of 50°. But, as in the tropical climate, a portion of caloric answering to 17° of Fahr. only is required to preserve the body at the natural temperature, it will be readily admitted that, if our account of the matter be correct, this evolution of superfluous caloric must exert no slight influence over the vital functions, and can hardly fail of being productive of danger to the constitution. It is, in all probability, to this increased evolution, and consequent accumulation of heat in the interior of the body, that we are to attribute the phlogistic diathesis, perhaps *plethora-ad-volumen*, increased thirst, diminished appetite, scanty excretion of high-coloured urine, and, in short, that general derangement of the constitution, so observable in the persons of northern strangers, which, if not fever, is, at least, very analogous to fever." In support of these conclusions, the author refers to the ingenious speculations of Dr. Bancroft, and others.

"To guard against these evils, nature, it is true, excites a more copious cuticular discharge; but, on account of the rigidity of fibre incident to strangers, this compensating influence is much less felt by them, than by the natives, or long residents."

"On these principles, we can readily explain the increased tendency to acute diseases, so observable in Europeans on their first arrival in a tropical climate,—leaving entirely out of view another powerful and constantly operating cause, viz. the direct stimulus of external heat. It is to this other cause that we are to attribute that continued tendency to inflammatory diseases which obtains in Europeans, even then when we must believe the body has so far accommodated itself to the medium in which it is placed as only to produce the degree of caloric necessary to its healthy constitution. The temp. of the sun's rays varies from 120° to 130°, that of the air from 80° to 93°,—a degree of heat which can never be quite harmless to the natives of northern regions."

"This [to them] unnatural degree of heat stimulates the skin, weakens its texture, and excites an increased action in the small vessels throughout the whole surface of the body. In consequence of the well-known *sympathy* between the skin and all the viscera—particularly the abdominal—the external stimulus operates internally, and the extreme vessels of the abdomen, especially those of the vena portæ, assume a similar increase of action.* This unusual determination of blood towards these parts, thus produced and kept up for a greater or less time, augments the biliary secretion, and eventually produces chronic inflammation, and gradual increase of size in the liver itself. This view of the process is supported by the opinions of two excellent physicians, namely, Drs. Johnson and Sanders.†"

"Of the correctness or inaccuracy of the above reasoning we leave others to decide; of the fact, however, namely, that Europeans living in tropical climates are subject to great derangements of the biliary secretion; in other words, to biliary diseases, there can be no question;

* See Johnson on Tropical Climates, passim.

† See Saunders on the Liver, p. 157.—165.

its truth has been established by the testimony of universal experience."

"In respect of the cause of this disease, I agree with those writers who consider both dysentery and fever, as produced by the same miasm. And if we wish to ascertain the particular circumstances which at one time determine the same agent to produce fever, and at another dysentery,—it appears to me that this difference of operation may, with much probability, be attributed to differences in the season of the year, and the constitutions of those subjected to its influence. Perhaps also the particular disease may be determined by chance, or at least by circumstances to us unknown, and therefore unappreciable."

"In a hot and dry season the miasm appears usually to affect the heart and arteries,*—thus producing irregular determinations of blood akin to inflammation; and it would, moreover, seem, that, owing to the great heat of the sun at these times, this determination is more especially directed to the head. Of this peculiar influence of the solar heat in causing an increased flow of blood towards the head, we have an obvious instance in the headach and nausea excited in those persons who expose themselves uncovered, even for a very short time, to the beams of a tropical sun.† "Hence we can account for the ardent fever appearing epidemically chiefly in the driest and most cloudless season of the year."

"In the rainy season, on the contrary, when the air is not only hot but moist, the miasmal poison seems more disposed to attack the abdominal viscera, predisposed as they have already become to acute diseases. The humidity of the atmosphere diminishes, and in some degree obstructs, the due discharge by the skin (for the moisture of the ambient air prevents rapid evaporation;)—the equilibrium of the circulation is thereby disturbed; the healthy consent or mutual sympathy between the skin and bowels is deranged; and, consequently, the unusual

---

* Although it appears evident that all miasmata act primarily on the nervous system, yet, in general we are unable to observe this primary operation, and for the most part remain unsuspicious of their deleterious influence till manifested in the derangements of the vascular system.

† Is this quite a fair deduction of our author? May not the nausea and headache in this case, be produced by *other causes* besides an increased flow of blood to the head,—by causes, for example, operating *immediately* through the medium of the nervous system? *Rev.*

determinations and congestions of blood, produced by the action of the poison, are directed to the abdominal viscera rather than to the cerebral mass. It thus happens, I conceive, that Dysentery is the prevailing disease at this period of the year; and it was, no doubt, from his having observed this fact, that we find Sydenham speaking of "*the ordinary fever of the season turned in upon the intestines.*" See Sydenhami Opera, p. 102—530—533.

"From a consideration of these circumstances, it will readily be understood, how, in any given number of men, in the same ship and at the same time, some shall be attacked with the pure endemic fever, and some with Dysentery;—the operation of the same miasm, namely, producing in some congestion in the brain,* in others in the abdominal viscera, according to the peculiar state of individual constitutions at the time."

"While off the Mississippi I never saw an attack of Dysentery (with the exception of a very few sporadic cases) but in the persons of those men, who, from having been employed on the shore or in the boats on the Lakes and River Apalachicola, and sleeping on its banks, had been frequently, and for a considerable length of time, exposed to the influence in question. As, however, of the men thus exposed, some were seized with ardent fever and some with Dysentery—I was more and more convinced of the identity of cause in both. Against the correctness of this opinion Dr. Johnson, with his usual ingenuity, has advanced the following argument:

"If," says he, "the *remote* cause of Dysentery, as well as of Yellow Fever, must be sought in *Marsh Miasmata,* how comes it to pass, that the same period of time which secures us from their operation in the one case (fever) should render us peculiarly liable to it in the other? This is surely blowing hot and cold with the same breath!" P. 25.

The following seems to me an explanation of this difficulty:—Since Dysentery is owing to unnatural conges-

* It appears from this and other expressions of our author in this Section, that he takes the doctrine so powerfully advocated by Dr. Clutterbuck,—viz. of fever being always and essentially inflammation of the brain,—as if fully and satisfactorily established. We are, however, by no means ready to concede this point to him; and certainly think, that it would have been sufficient to state that he himself was a convert to the doctrine, without presuming that all the world was of the same opinion. Rev.

tion of blood in the liver, or, at least, to the consequences of this — inflammation and depraved biliary secretion,— it is not surprising that it should recur more than once; as we know that this gland, like the tonsils, testicles, &c. after an attack of inflammation, is prone to similar affections. How the preceding attacks predispose to others we cannot certainly say; but it appears, at least, that they produce a change of structure as well as an augmentation of size, both of which seem favourable to future inflammatory action.—The case is very different with endemic fever.—In it, although there be increased determination of blood to, and consequent congestion in, the brain, these produce no permanent change of structure (except in fatal cases), and therefore leave no *structural* derangements to favour the inroads of future disease; and it is a well-known fact, that the true phrenitis, or inflammation of the brain, does not predispose to the same complaint. On this account, then, it may happen, that, although, after long residence in warm climates, the same species of miasm may still possess the power to produce either *fever* or *dysentery* as before,—yet, from the reasons just enumerated, and on account of the great sympathy of the liver with the skin, it will rather produce the latter than the former disease.

On all occasions it is unquestionably a very difficult matter truly to ascertain the proximate causes of disease; in the present case, however, I have little hesitation in considering this disease, (Dysentery) in its first stage, to be nothing more than an increased action of all the abdominal vessels, and particularly of the venæ portæ, tending to, and in most cases ending in, a chronic inflammation of the liver. How long the disease may remain in this its primary masked form, before it exhibits a marked degree of pyrexia and other more characteristic symptoms of Dysentery, I cannot certainly say; the period may perhaps vary from two to four or eight days, according to circumstances: in all cases, however, whether the period be long or short, that slow and insidious pyrexia already described, uniformly accompanies the early progress of the affection. The liver gradually becomes enlarged; its proper functions become more and more deranged, and, at length, instead of a healthy bile, there is formed a fluid different both in quantity and quality from the natural secretion; which fluid acts upon the intestines as a virulent stimulus, and eventually occasions the ulcerations of their tunics, and the violent accompanying symptoms above detailed. This fact has been noticed by Dr. Saunders." *

"From all these considerations, I am of opinion that the Dysentery of hot climates derives its origin from a miasm, and is to be considered as a fever attended by a topical affection of the abdominal viscera, and particularly of the liver. It has been imagined, that the dark-coloured blood observed in the stools of dysenteric patients is derived from the ulcerations of the intestinal tunics; it appears to me, however, much more probable, that the greater part, at least, of this comes from the liver,—"ex fonte uberiore scateat." I imagine that the blood, on account of its increased impetus through the torpid vessels of that viscus, is forced unchanged from the minute ramuli of the venæ portæ into the pori biliarii, and thence is conveyed, along with the bile, into the alimentary canal. The very dark colour of the blood ejected by dysenteric patients favours, in no slight degree, this opinion of its origin,—the superior intensity of blackness in the portal blood being well known."

"That *true idiopathic Dysentery* (that is, Dysentery unattended by morbid alterations of other viscera besides the intestines,) may exist between the Tropics I will not deny, since practical authors inform us, that they have seen it;—or, at least, in their descriptions of this disease make no mention of the hepatic affection. But, I think, I can safely assert, that no case presented itself to me in the expedition to New Orleans, that was not complicated with a diseased state of the liver. Nor has this opinion been adopted rashly or on slight grounds. The dissection of the bodies of those that fell victims to the disease,† as well as the nature of the remedies found most efficacious in curing it, indeed sufficiently convinced me not only that the dysenteric flux was complicated with a diseased state of the liver, but that the greater part of its violent and dangerous symptoms originated essentially in this

* See Saunders on the Liver, p. 183—332.

† "Abscesses, as I have mentioned above, were generally found in the substance of the liver. In the cases where pus was not detected, the tissue of the hepatic vessels was so much altered by the effects of former inflammation and congestion, that the gland was so tender as to yield to a very slight pressure of the finger. I remarked this condition of parts in those who had laboured under the disease accompanied with a fever of the remittent type."

organ; and I was taught, by too fatal and extensive experience, that it was vain to hope for the cure of the intestinal symptoms until that organic derangement in which they had their origin was previously removed."

"As the disease I have been describing is so obviously allied to Hepatitis, the name *Dysentery,* used in this Essay, has given offence to some very adequate judges.* This, however, seeing that I am not the inventor of the name, nor the first that applied it to the disease in question, gives me very little concern;—particularly as the nomenclature, in the present case, can lead to no practical error. Besides, I am of opinion, that in every severe case of flux in tropical climates, the liver *is* always sooner or later affected.—By what title then (it may be asked) shall we designate this too frequent and too fatal disease, if, on account of the accompanying affection of the liver, we reject the common name of *Dysentery?*"

The author then makes some observations in disproof of its supposed contagious nature, and then concludes this section thus:

"Hanc Symptomatum rationem utcûnque mancam et inconditam, eruditis judicibus meis subjicere volo; indulgentiâ eorum, potiùs quàm viribus propriis, inniti paratus. An opinio nunc in medium prolata, justa sit, an secùs, judicent periti—testetur experientia futura."

The author then gives, in very few words, the diagnosis between Dysentery and those diseases with which it is most likely to be confounded—viz. Cholera, Diarrhœa, Enteritis, and Cholic. It seems rather singular that he has not in this place taken any notice of Hepatitis properly so called. Surely he does not mean to assert, that the disease he has described under the name of "Dysentery of Hot Climates," is really one and the same disease as the Oriental Hepatitis; if not,—we think it is not a little remarkable that he has not once mentioned *Hepatitis* in his short section "De Diagnosi."

In the section devoted to the *Cure* of Dysentery, the author briefly details the proper use and administration of the following remedies—viz. Bandaging the abdomen with flannel;—mild cathartics;—warm bath;—diaphoretic anodyne draughts;—plentiful use of warm mild mucilaginous liquors;—blood-letting. Of the utility of this last remedy he speaks in pretty strong terms, and as-

* See London Medical and Physical Journal, May 1816.

sures us, that he has found its repeated employment most advantageous in this disease. We give our strong assent to this part of Dr. R's practice, and cannot help thinking that, if V. S. had been still more liberally had recourse to than it appears to have been by him, there would have been less occasion for the grand and highly-lauded specific, *Calomel*. In justification of this our opinion, we beg simply to refer to the history of the disease, and the appearances on dissection, as given by Dr. R. himself.

But in our author's opinion all these remedies are of very secondary importance:

"Hæc autèm inutilia, et ad morbi formam graviorem pacandam parùm valentia, experientia infausta luculentèr docuit; et nisi ad medicamen fortius et certius gnavitèr confugiatur, morti ægrum eripere medicus vix poterit."

This "more potent and surer remedy" is Calomel, in the exhibition of which Dr. R. appears to have been guided by the details of practice contained in Dr. Johnson's book on Tropical Climates. He gave it in *scruple doses* twice a day, combined with a grain and a half of opium; and assures us that hypercatharsis, or, indeed, any other bad effects, need not be apprehended from this practice. He advises it to be continued until the supervention of ptyalism, as, he says, it is not by its cathartic powers, but by its specific action on the system, that it operates in curing the disease. Its general influence in combating this dreadful malady is thus summed up by Dr. R.

"Actionem inflammatoriam vasorum jecinoris tollit, ductus onere bilis acris gravatos emulgit, secretionem hepatis naturalem suscitat, pyrexiam solvit, et intestina evacuat. Hoc remedium a Doctore Curry *Cholagogus* appellatur, et sanè nomen optimè meruit. Sine dubio praestat conatus nostros contra radicem morbi dirigere, remedium dando unicum et simplex, quàm aegrum per plures dies vexare, purgantia et anodyna alterna, exhibendo. Submurias Hydrargyri vix nimìs laudari potest: simùl ac virtutes suas speciales in corpus effecerit, profluviumque salivae excitaverit, indicia morbi omnia, saepè noctis unius spatio, suâ sponte abibunt, et faeces colorem flavum magìs magìsque naturalem recuperabunt. Hic color dejectionum, quantùm adhùc observavi, signum morbi pacati optimum et certissimum est. Tùnc nil actu necessarium erit nisi redintegrare vires ventriculi usu decocti Quassiae Excelsae, Corticis Peruviani vel similium, ventrem indentidèm leni purgatione laxare, quià, dum fluit saliva, interdùm adest obstipatio!—res sanè haud parùm insignis."

After this ample detail,—into which we have been led, partly by the peculiar nature of the publication, partly by sweet and bitter association, with former scenes and sufferings,—but chiefly from a belief that we were ministering to the gratification of our readers,—we cannot occupy any more of our pages with this subject. Dr. R. perhaps may congratulate himself on the circumstance; as, certainly, there are several doctrines and opinions advanced by him, which, we flattered ourselves while perusing, we should enjoy the rapture of critics — *certaminis gaudia* —in confuting and overturning. The loss of this happiness we shall however the less regret on the present occasion, as we feel that opinions promulgated through the channel of inaugural academical dissertations,—when the author can honestly say with Dr. R. " haud quaquam novitatis illecebris, aut vanæ laudis amore, in hoc penso suscipiendo, perculsus sum,"—we say, in these cases, opinions more hetorodox than any of our author's will always find in us rather indulgent judges than cynical censurers.

Dr. R. however, needs not apprehend the fiat of the most impartial and rigid tribunal;—and before finally taking leave of him, we venture to express a hope, that the talents and elegant learning displayed in this initiatory essay may hereafter claim the exercise of our judicial functions by labours if not of greater importance, at least of higher pretensions.

We shall indulge ourselves with one more quotation as a specimen of the elegance of the stile, and classic air of the latinity, of this little volume: it is the commencement of the " Historia Morbi."

" Inter criminum catervam e pravis generis humani moribus exorientium, bella probis detestata, in ordine primo ritè et legitimè collocanda sunt. Sed quo infestiora et insigniora sint crimina, eo pejus et insignius supplicium auctoribus suis inferre solent, quia (secundum Justitiae Sempiterane statuta,) " Sceleris est sese punire." Hinc Bellum adeo casibus opimum, et miseriae omnigenae mole gravidum, tantam morborum prolem facili nixû semper peperisse, videretur.

Militia sanè nusquàm malorum hujusmodi expers, sed inter sequelas ex hoc fonte sanguinolento provenientes, Dysenteriam potissimùm eminere, omnibus qui rei militari sese dederint, sine ope verborum luculentèr patebit. Castris et classibus bellicis, velùt sedi ejus speciali ac solio exoptato insidet, sceptrum invisum populo querenti tendit, atque imperium moestum, nisi cum morte, dividere non-vult. Formam sibi induere solet quàm maximè violentam, nunqùam sanè, vel raró saltèm, alibi visam: namque hominibus procul bello degentibus, artes Pacis mutas mitesque agitantibus, et commodis vitae modicis fruentibus, clades ejus exitiosas vix, ac ne vix quidem, experiri contigit.

Quamobrem Morbus Classium et Castrorum paritèr ac Comes et Castigator, κατ' ἐξοχὴν appellari potest."

